# Ectopic Expression of a *Thellungiella salsuginea* Aquaporin Gene, *TsPIP1;1*, Increased the Salt Tolerance of Rice

**DOI:** 10.3390/ijms19082229

**Published:** 2018-07-30

**Authors:** Wei Li, Xiao-Jing Qiang, Xiao-Ri Han, Lin-Lin Jiang, Shu-Hui Zhang, Jiao Han, Rui He, Xian-Guo Cheng

**Affiliations:** 1 Lab of Plant Nutrition Molecular Biology, Institute of Agricultural Resources and Regional Planning, Chinese Academy of Agricultural Sciences, Beijing 100081, China; 11714057@zju.edu.cn (W.L.); danajiang@163.com (L.-L.J.); 2 Institute of Environment and Sustainable Development in Agriculture, Chinese Academy of Agricultural Sciences, Beijing 100081, China; qiangxiaojing11@163.com; 3 College of Land and Environment, Shenyang Agricultural University, Shenyang 110866, China; hanxiaori@163.com (X.-R.H.); 13039514944@163.com (S.-H.Z.); 13051377892@163.com (R.H.); 4 College of Life Science, Shanxi Normal University, Linfen 041004, China; hanjiao19910905@126.com

**Keywords:** *TsPIP1;1* gene, aquaporin, differentially expressed genes, salt stress, transgenic rice

## Abstract

Aquaporins play important regulatory roles in the transport of water and small molecules in plants. In this study, a *Thellungiella salsuginea TsPIP1;1* aquaporin was transformed into Kitaake rice, and three transgenic lines were evaluated by profiling the changes of the physiological metabolism, osmotic potential, and differentially expressed genes under salt stress. The TsPIP1;1 protein contains six transmembrane domains and is localized in the cytoplasm membrane. Overexpression of the *TsPIP1;1* gene not only increased the accumulation of prolines, soluble sugars and chlorophyll, but also lowered the osmotic potential and malondialdehyde content in rice under salt stress, and alleviated the amount of salt damage done to rice organs by regulating the distribution of Na/K ions, thereby promoting photosynthetic rates. Transcriptome sequencing confirmed that the differentially expressed genes that are up-regulated in rice positively respond to salt stimulus, the photosynthetic metabolic process, and the accumulation profiles of small molecules and Na/K ions. The co-expressed *Rubisco* and *LHCA4* genes in rice were remarkably up-regulated under salt stress. This data suggests that overexpression of the *TsPIP1;1* gene is involved in the regulation of water transport, the accumulation of Na/K ions, and the translocation of photosynthetic metabolites, thus conferring enhanced salt tolerance to rice.

## 1. Introduction

High soil salinity is a serious ecological obstacle factor limiting agricultural production and crop yield [1]. Usually, salt stress triggers physiological damage in plants and leads to a series of alterations in photosynthesis, ion flux, and water transport [2]. Plant aquaporins (AQPs) play important regulatory roles in the adaptive acclimation to physiological changes in plants in response to salt stress and are widely present in living organisms [3]. AQPs have been confirmed to be involved in the symplast pathway that efficiently dominates the transmembrane transport of water in plants and have demonstrated a specific structure covering six transmembrane domains with two conserved Asn-Pro-Ala (NPA) motifs [4].

Previously, plant *AQP* genes have been functionally characterized under environmental stress. The *TrPIP2;1* gene in *Tamarix ramosissima* plays an important regulatory role in enhancing foliar water uptake [5]. Overexpression of the *OsPIP1;3* gene could maintain water balance in transgenic plants by interacting with the OsPIP2 subfamily in response to chilling stress [6]. The tobacco *NtAQP1* gene promotes the transmembrane diffusion of CO_2_ by increasing the net photosynthesis and mesophyll conductance rates in tobacco [7]. Phosphorylation of the AtPIP2;1 protein at Ser283 obviously accommodates water permeability in the cell membrane by decorating the phosphorylation site under salt stress [8]. NOD26, a transmembrane channel protein, remarkably improves the transport of water and glycerol and participates in osmotic regulation during the symbiosis of soybeans and rhizobia [9].

Although the *TsTIP1;2* gene acts as a multifunctional contributor to increase the tolerance of the transgenic *Arabidopsis* under highly stressful conditions [10], its regulatory role in *Thellungiella salsuginea* remains unclear. Studies have shown that *Thellungiella salsuginea* displays a strong resistance to high salinity, drought, and chilling [11]. Therefore, we isolated an aquaporin *TsPIP1;1* gene from *Thellungiella salsuginea* that is distinct from other higher plant species, transformed it into the Kitaake *Oryza sativa* L. ssp. japonica rice cultivar by the *Agrobacterium*-mediated method, and obtained T3 generation transgenic rice by continuously screening. Three transgenic lines with stable heredity, OE-17, OE-18, and OE-19, were randomly selected and specifically cultured by the Murashige and Skoog (MS) solid medium or hydroponics under salt stress, and changes in the physiological metabolites, osmotic potential, accumulation of K and Na ions, flux of K and Na ions, photosynthetic parameters, and expression profiles of the target genes in the transgenic rice were systematically investigated. Meanwhile, analyses of transcriptome sequencing confirmed the involvement of up-regulated differentially expressed genes in the physiological metabolism and photosynthetic processes of transgenic rice in response to salt stress. Therefore, our results suggested that the overexpression of the *TsPIP1;1* gene enhances the salt tolerance of rice by maintaining osmotic potential and promoting photosynthesis.

## 2. Results

### 2.1. Analyses of the TsPIP1;1 Gene’s Structural Characteristics

**Figure 1:**
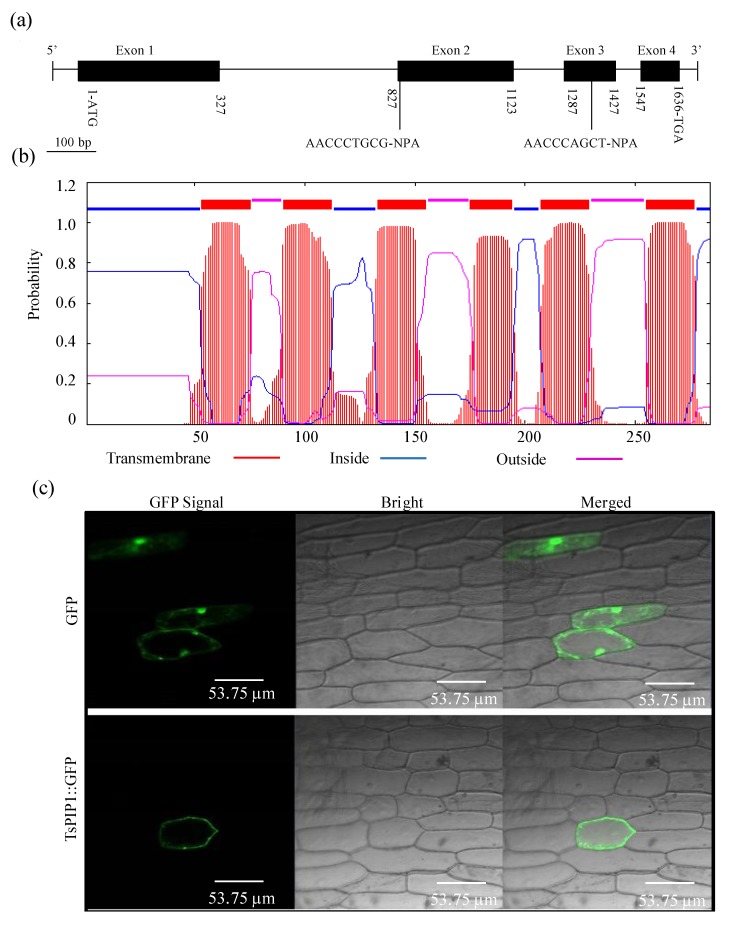
The structural profiles and subcellular localization of the *TsPIP1;1* gene. (**a**) The structural composition of the *TsPIP1;1* gene. (**b**) Prediction of transmembrane helices of the TsPIP1;1 protein. (**c**) Subcellular localization of the TsPIP1;1 protein in the onion epidermal cells.

The bioinformatic analysis showed that the *TsPIP1;1* gene is composed of four exons and three introns and encodes 284 amino acids. Four exons are represented by four fragments containing 327-, 297-, 141-, and 90 bp, respectively, in which the second and the fourth exon fragments contain a conserved NPA (Asparagine-Proline-Alanine) motif (Figure 1a). Structural prediction showed that the TsPIP1;1 protein has six transmembrane helices, and both the N- and C-termini of the TsPIP1;1 protein are located in the cytoplasm (Figure 1b), which demonstrates the typical structural characteristics of a major intrinsic protein. Subcellular localization showed a haploid strong green fluorescent protein (GFP) signal at the cytoplasm membrane in the onion epidermal transformants of the *TsPIP1;1::GFP* construct, indicating that the TsPIP1;1 protein is specifically located in the cytoplasm membrane in onion epidermal cells (Figure 1c).

### 2.2. The Phenotypic Characteristics of Transgenic Rice under Salt Stress

**Figure 2:**
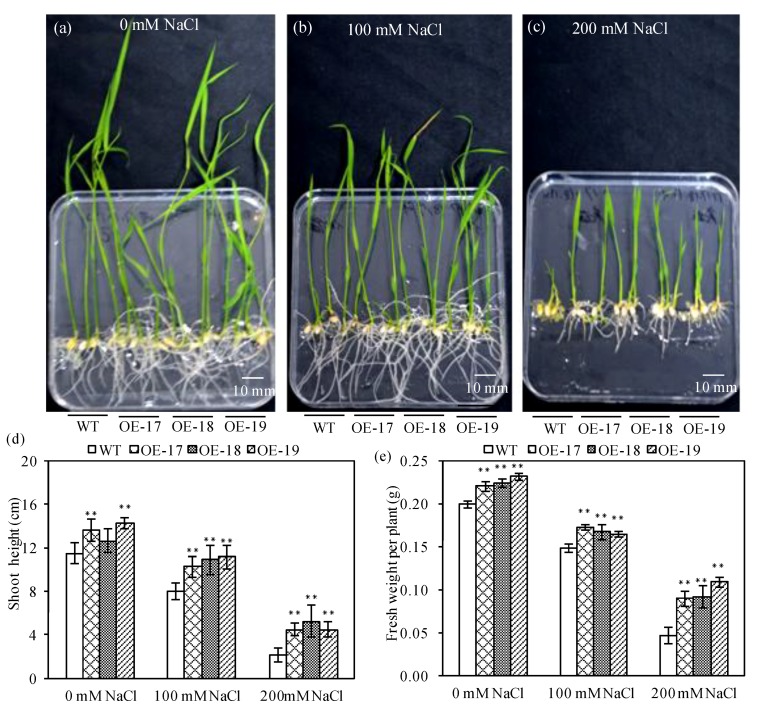
The phenotypic characteristics of Murashige and Skoog (MS) solid medium-cultured transgenic rice under salt stress. Three treatments are shown: (**a**) 0 mM NaCl (**b**) 100 mM NaCl and (**c**) 200 mM NaCl. (**d**) Shoot heights. (**e**) Fresh weight per plant. The error bars represent the ± SD of the three biological replicates; ** indicates a significant difference at the *p* < 0.01 level, respectively (*LSD*-test). WT represents wild-type; OE-17, OE-18, and OE-19 accordingly represent the transgenic rice lines.

The morphological changes of rice under salt stress and favorable conditions were separately photographed (Figure 2a). Under favorable culture, both the transgenic and wild-type rice demonstrated better growth on the MS solid medium, but salt stress seriously inhibited rice growth. In the case of salt stress, the transgenic rice revealed better phenotypes than the wild-type (Figure 2b,c) and an increased biomass, especially in the case of salt stress of 200 mM of NaCl. The data show that both the average shoot heights and fresh weights of the transgenic OE-17, OE-18, and OE-19 lines were significantly higher than those of the wild-type (Figure 2d,e).

**Figure 3:**
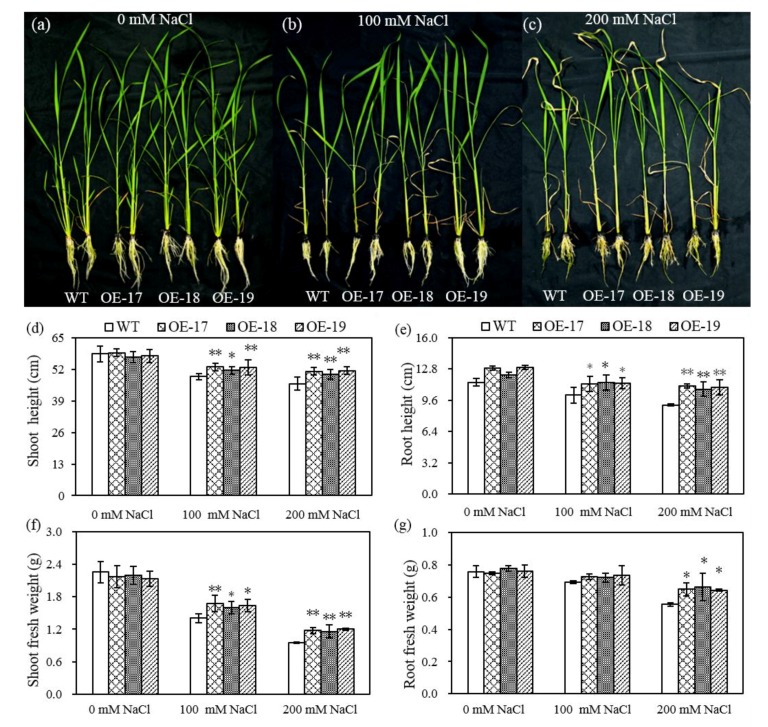
The differences in phenotypes and in the biomass of rice in response to NaCl stress. The rice phenotypes were placed in hydroponics with solutions of 0 mM NaCl, (**a**) 100 mM NaCl, (**b**) and 200 mM NaCl (**c**). (**d**) Shoot height. (**e**) Root height. (**f**) Shoot fresh weight. (**g**) Root fresh weight. The others are the same as in Figure 2. * and ** indicate significant differences at the *p* < 0.05 level and *p* < 0.01 level, respectively (*LSD*-test).

The hydroponics experiment also showed similar results in that both the transgenic rice and the wild-type had no significant differences in phenotypic changes under favorable culturing (Figure 3a). However, salt stress remarkably led to the leaf withering of rice and the decrease in biomass (Figure 3b,c). The transgenic lines seemed to show more green leaves and revealed stronger salt resistance than the wild-type in response to salt stress. Especially in the case of salt stress with 200 mmol·L^−1^ of NaCl, both the shoot heights and fresh root weights of the transgenic rice were significantly higher than those of the wild-type (Figure 3d–g).

### 2.3. Relative Expressions of the TsPIP1;1 Gene and Physiological Changes

**Figure 4:**
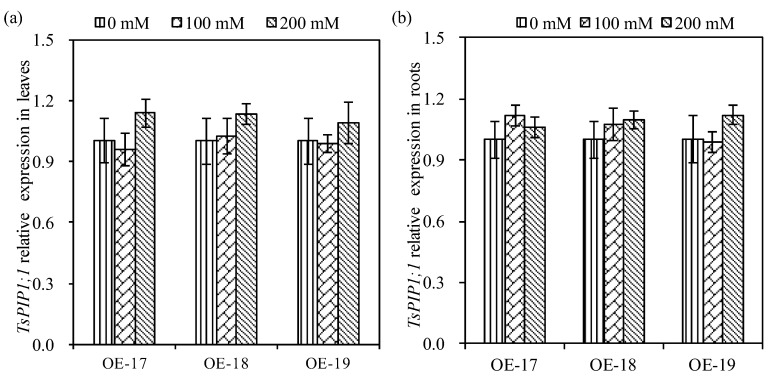
The expression profiles of the *TsPIP1;1* gene in transgenic rice under salt stress. Relative expression of *TsPIP1;1* in the leaves (**a**) and roots (**b**).

To profile the *TsPIP1;1* gene’s expression in the transgenic rice, RT-qPCR was performed. The data shows that the expression of the *TsPIP1;1* gene in both the leaves and the roots were not repressed by salt stress (Figure 4). Three transgenic lines, OE-17, OE-18, and OE-19, accumulated more transcripts of the *TsPIP1;1* gene in leaves under a salt stress of 200 mmol·L^−1^ of NaCl (Figure 4a). Both the transgenic OE-17 and OE-18 lines demonstrated more transcripts of the *TsPIP1;1* gene in the roots under salt stress, but the transcripts of the *TsPIP1;1* gene in the roots of the transgenic OE-19 line increased relatively under salt stress with a solution of 200 mmol·L^−1^ of NaCl, although no significant differences were observed (Figure 4b).

**Figure 5:**
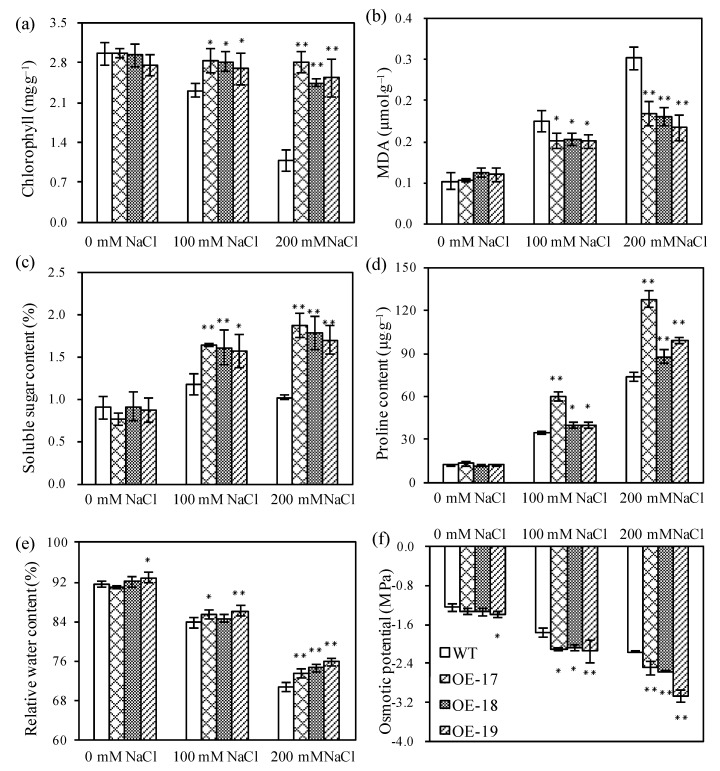
The accumulation profiles of physiological metabolites in rice lines in response to salt stress. (**a**) Chlorophyll content. (**b**) Malondialdehyde (MDA) content. (**c**) Soluble sugar content. (**d**) Proline content. (**e**) Relative water contents. (**f**) Osmotic potential of leaves. * and ** indicate significant differences at the *p* < 0.05 level and at the *p* < 0.01 level, respectively (*LSD*-test).

Most transcripts of aquaporins in plants have positive regulatory roles in promoting the transport of water and small molecular metabolites, which are usually closely associated with the acclimation of plant adaptability to salt stress. Therefore, we measured the accumulation of those metabolites related to the osmotic potential in rice. The data shows that the transgenic lines had no significant differences in the accumulation of chlorophyll, soluble sugar, proline, and malondialdehyde compared with the wild-type under favorable conditions (Figure 5). However, the transgenic lines accumulated a significantly higher amount of chlorophyll in response to salt stress compared to the wild-type. In the case of culturing with a solution of 200 mmol·L^−1^ of NaCl, the chlorophyll contents in the OE-17, OE-18, and OE-19 lines were increased by 2.6-fold, 2.3-fold, and 2.4-fold compared to the wild-type, respectively (Figure 5a). Figure 5b shows that MDA accumulation in the transgenic lines significantly decreased compared to the wild-type under salt stress. When the plants were exposed to the solution of 100 mmol·L^−1^ of NaCl, the accumulation of MDA in the leaves of the transgenic OE-17, OE-18, and OE-19 lines lowered by 0.039, 0.036, and 0.040 µmol·g^−1^, respectively. After the plants were cultured under 200 mmol·L^−1^ of NaCl, the transgenic OE-17, OE-18, and OE-19 lines were lowered by 0.107, 0.113, and 0.135 µmol·g^−1^ of MDA in the leaves compared to the wild-type, respectively. When the plants were exposed to high salinity, the soluble sugar and proline contents in the transgenic lines were obviously higher than those in the wild-type (Figure 5c). When the plants were cultured in a solution of 100 mmol·L^−1^ of NaCl, the soluble sugar content in the OE-17, OE-18, and OE-19 lines increased by 1.39-fold, 1.37-fold, and 1.34-fold compared to the wild-type, respectively. Under culturing with a solution of 200 mmol·L^−1^ of NaCl, the contents of the soluble sugar in the OE-17, OE-18, and OE-19 lines increased by 1.86-fold, 1.75-fold, and 1.67-fold compared to the wild-type, respectively (Figure 5c). When the plants were cultured in the solution of 100 mmol·L^−1^ of NaCl, the proline content in the OE-17, OE-18, and OE-19 lines increased by 1.72-fold, 1.14-fold, and 1.16-fold compared to the wild-type, respectively (Figure 5d), and the proline content in the OE-17, OE-18, and OE-19 lines increased by 1.73-fold, 1.19-fold, and 1.35-fold compared with the wild-type under stress with a solution of 200 mmol·L^−1^ of NaCl, respectively (Figure 5d).

To assess the change in osmotic potential in rice, both the relative water content in leaves and the osmotic potential of sap in shoots were measured. The data shows that compared to the wild-type, the relative water contents in the transgenic OE-17 and OE-19 lines increased by 1.61% and 2.33% after stress with 100 mmol·L^−1^ of NaCl, and by 2.71%, 3.84%, and 5.08% after stress with 200 mmol·L^−1^ of NaCl, respectively (Figure 5e). The results show that only the transgenic OE-19 line had obviously lowered the osmotic potential of the leaves under favorable conditions, but three transgenic lines had significantly lowered osmotic potentials in the leaves compared to the wild-type after being cultured with a solution of 100 mmol·L^−1^ of NaCl. In the case of culturing with a solution of 200 mmol·L^−1^ of NaCl, the average osmotic potential of the leaves of the three transgenic lines showed decreases of 0.33, 0.39, and 0.90 MPa compared to the wild-type, respectively (Figure 5f).

### 2.4. The Accumulation Profiles of K^+^ and Na^+^ in Transgenic Rice

The accumulation profiles of Na and K in plants have a close relationship with the osmotic potential, thus affecting the ion balance in plants. Table 1 shows that when plants were exposed to a solution of 200 mmol·L^−1^ of NaCl, the transgenic lines apparently lowered the Na content and increased the K content in the shoots compared to the wild-type, although both the transgenics and the wild-type demonstrated greater accumulation of Na and K in the shoots. The transgenic lines, in particular, seemed to accumulate more K in the shoots in response to salt stress compared with the wild-type. The contents of K in the transgenic OE-17, OE-18, and OE-19 lines were increased by 1.27-fold, 1.48-fold, and 1.49-fold compared with the wild-type, respectively (Table 1). The Na/K ratios in the transgenics were significantly lower than those in the wild-type after a salt stress of 200 mmol·L^−1^ of NaCl, indicating that the transgenics accumulated more K ions after salt stress (Table 1). The data shows that the Na/K ratios in the transgenic OE-17, OE-18, and OE-19 lines decreased by 0.28, 0.30, and 0.21 compared to the wild-type, respectively, and were significantly lower than 1.68 in the wild-type after treatment with a solution of 100 mmol·L^−1^ of NaCl. Meanwhile, the Na/K ratios in the transgenic OE-17, OE-18, and OE-19 lines decreased by 1.55, 2.03, and 2.09 relative to 5.35 in the wild-type after treatment with the solution of 200 mmol·L^−1^ of NaCl, respectively (Table 1).

### 2.5. The Flux Analyses of Na and K in the Roots of Transgenic Rice

**Figure 6:**
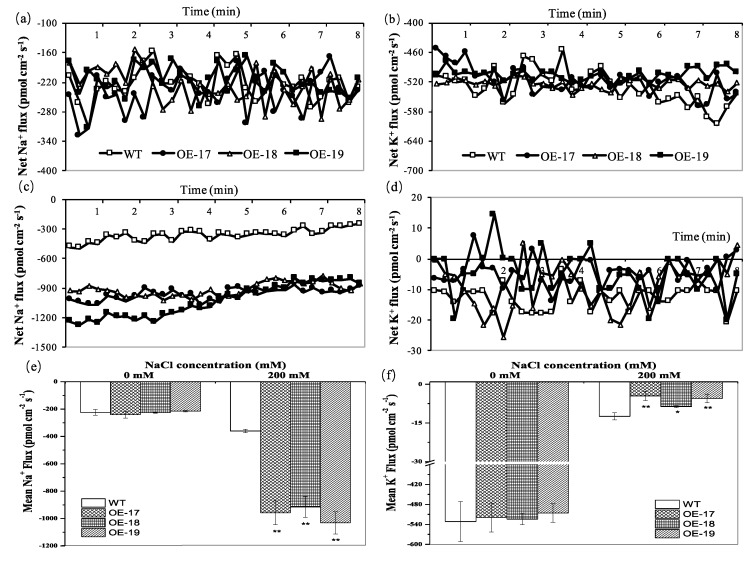
The flux profiles of Na^+^ and K^+^ at the root tip elongation zones of 7-day-old seedlings. (**a**) The net Na^+^ flux without NaCl. (**b**) The net K^+^ flux without NaCl (0 mM NaCl). (**c**) The net Na^+^ flux with a supply of 200 mM NaCl. (**d**) The net K^+^ flux with a supply of 200 mM NaCl. The net effluxes are indicated by negative values and the net influxes are indicated by positive values. (**e**) The mean Na^+^ flux. (**f**) The mean K^+^ flux. * and ** indicate significant differences at the *p* < 0.05 level and *p* < 0.01 level, respectively (*LSD*-test).

To characterize the net fluxes of Na and K ions at the surface of the roots, we measured the flux rates of Na and K ions in the root tips of 7-day-old rice seedlings in 8 min by the non-invasive micro-test technique (NMT). The results show that when the seedlings were exposed to favorable culture solutions, both the transgenic lines and the wild-type exhibited significant fluctuation in the Na ion flux in the elongation zone of the root tips (Figure 6a), and the K ion flux in the elongation zones exhibited little fluctuation (Figure 6b). However, when the rice seedlings were exposed to a solution of 200 mmol·L^−1^ of NaCl, both the transgenic lines and the wild-type showed gradual decreases in Na ion efflux with time extension, but the transgenic line significantly increased the Na ion efflux in the elongation zones of the root tips compared to the wild-type (Figure 6c). The flux of the Na and K ions in the elongation zones exhibited unstable fluctuations at different measurement points in response to salt stress. The data show that the transgenic line obviously decreased the efflux of the K ion in the root tips compared to the wild-type although the transgenic lines root tips revealed influx of the K ion at some measurement points (Figure 6d). When the rice was cultured under favorable conditions, the net flux of both Na and K ions in the wild-type and the transgenic lines had no significant differences (Figure 6e). However, when the rice seedlings were exposed to the solutions of 200 mmol·L^−1^ of NaCl, the average net influxes of the Na ion in the root tips of the three transgenic OE-17, OE-18, and OE-19 lines were accordingly lowered by 2.7-fold, 2.5-fold, and 2.9-fold compared to the wild-type (Figure 6e), while the average net influxes of the K ion in the root tips of the OE-17, OE-18, and OE-19 lines increased by 2.7-fold, 1.4-fold, and 2.3-fold compared to the wild-type, respectively (Figure 6f).

### 2.6. Transgenic Rice Overexpressing the TsPIP1;1 Gene Showed Enhanced Photosynthesis

To profile the salt stress-triggered photosynthetic changes, we measured the photosynthetic parameters in the rice leaves under salt stress (Table 2). The data showed that the three transgenic OE-17, OE-18, and OE-19 lines had significantly improved photosynthetic rates in the leaves in response to salt stress compared to the wild-type. Similar to photosynthetic rates, both the stomatal conductance (Gs) and intercellular CO_2_ concentration (Ci) in the leaves of the transgenic lines were significantly higher than those in the wild-type. Under a favorable culture and salt stress of 100 mmol·L^−1^ of NaCl, only the OE-19 significantly enhanced the transpiration rate (Tr), but the three transgenic lines significantly promoted transpiration rates under a salt stress of 200 mmol·L^−1^ of NaCl compared to the wild-type. The water use efficiency (WUE) in the transgenic lines increased by 0.14, 0.25, and 0.24 μmol·mmol^−1^ under a salt stress of 100 mmol·L^−1^ of NaCl, and by 0.14, 0.12, and 0.28 μmol·mmol^−1^ under a stress of 200 mmol·L^−1^ of NaCl, compared with the wild-type, respectively (Table 2).

**Figure 7:**
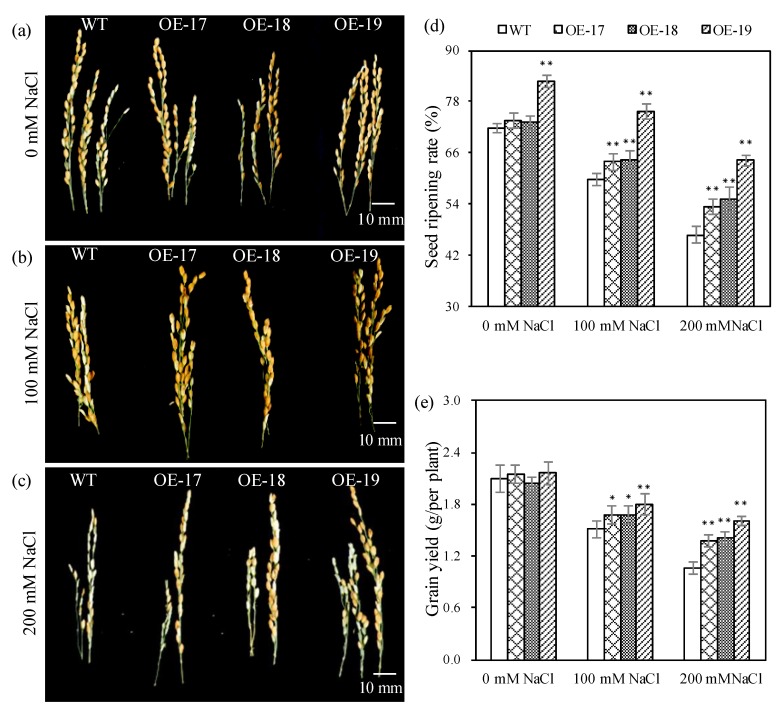
The profiles of the panicle and rain at the harvesting stage of rice. The grain phenotypes were exposed to 0 mM NaCl (**a**), 100 mM NaCl (**b**), and 200 mM NaCl (**c**); (**d**) Seed ripening rate. (**e**) Grains yield per plant. * and ** indicate significant differences at the *p* < 0.05 level and at the *p* < 0.01 level, respectively (*LSD*-test).

To understand the effects of photosynthesis changes on rice productivity, both seed ripening rates and grain yield per plant (*n* = 5) were investigated. Our data show that the transgenic lines had significantly raised seed ripening rates and grain yield per plant than the wild-type (Figure 7a–c). Under a salt stress of 200 mmol·L^−1^ of NaCl, the seed ripening rates in the transgenic OE-17, OE-18, and OE-19 lines increased by 6.68%, 8.47%, and 17.54% (Figure 7d), and the grain yield per plant increased by 0.32, 0.35, and 0.55 g, compared to the wild-type, respectively (Figure 7e).

### 2.7. Responses of Differentially Expressed Genes in the Transgenic Lines to Salt Stress

**Figure 8:**
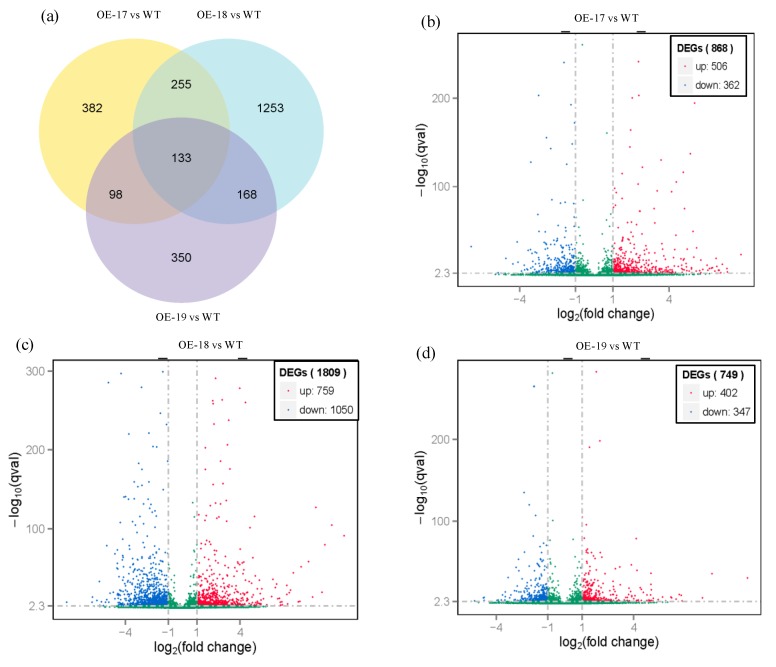
An overview of differentially expressed genes (DGEs) in rice in response to a stress of 100 mM NaCl. (**a**) Venn diagram of the DEG number. (**b**) Volcano distributions of up- and down-regulated DEGs in the OE-17 vs. the wild-type. (**c**) Volcano distributions of up- and down-regulated DEGs in the OE-18 vs. the wild-type. (**d**) Volcano distributions of up- and down-regulated DEGs in the OE-19 vs. the wild-type.

To profile the differentially expressed unigenes in the transgenic rice, both hierarchical clustering and most enriched GO (Gene Ontology) terms were performed, and the relative expression levels of the differentially expressed unigenes were normalized to log_2_ (fold change). The number of differentially expressed genes (DEGs) in transgenic rice is shown in a Venn diagram compared to the wild-type under a stress of 100 mmol·L^−1^ of NaCl (Figure 8a). Overlapping analyses show that three combinations—OE-17 vs. wild-type, OE-18 vs. wild-type, and OE-19 vs. wild-type—exhibited 133 co-expressions of differentially expressed unigenes. The number of up-regulated and down-regulated unigenes in the transgenic OE-17, OE-18, and OE-19 lines demonstrated remarkable differences. Under a salt stress of 100 mmol·L^−1^ of NaCl, the number of differentially expressed genes in the combination of OE-17 vs. WT was composed of 506 up-regulated and 362 down-regulated genes (Figure 8b); the combination of OE-18 vs. WT showed the up-regulation of 759 genes and the down-regulation of 1050 genes (Figure 8c); and the combination of OE-19 vs. WT revealed the up-regulation of 402 genes and the down-regulation of 347 genes (Figure 8d).

**Figure 9:**
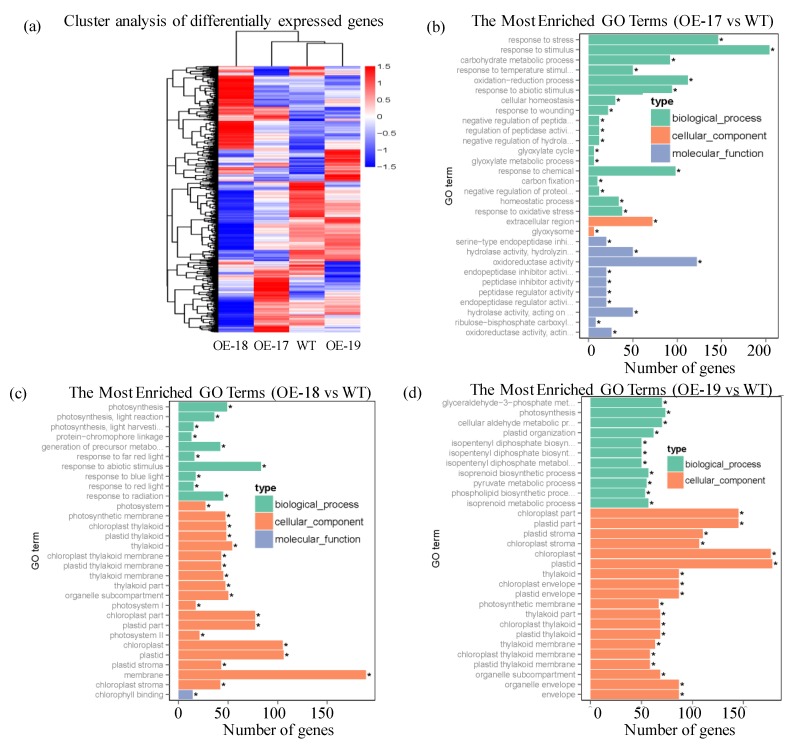
The distribution profiles of up- or down-regulated differentially expressed genes. (**a**) Cluster analysis of differentially expressed genes. (**b**) The most enriched GO terms of the up-regulated genes in the transgenic OE-17; (**c**) The most enriched GO terms of the up-regulated genes in the transgenic OE-18; (**d**) The most enriched GO terms of the up-regulated genes in the transgenic OE-19. * indicates significant differences at the *p* < 0.05 level.

A cluster analysis shows that the transgenic lines had substantial differences in the expression patterns of all unigenes compared with the wild-type in response to a salt stress of 100 mmol·L^−1^ of NaCl (Figure 9a). The hierarchical cluster of the differentially expressed unigenes was divided into 22 subclusters and revealed corresponding expression patterns of unigenes in the same subcluster, indicating that the three transgenic lines demonstrated different responsive mechanisms in expressing the unigenes in response to salt stress. As shown in Figure 9b, the most enriched GO terms of the up-regulated genes in the OE-17 line mainly responded to abiotic stimuli in the biological process. It also revealed the responses to hydrolase activity and oxidoreductase activity in molecular functions. In both the transgenic OE-18 and OE-19 lines, the up-regulation of differentially expressed genes were mainly related to physiological metabolism as well as the chloroplast, plastid, and membrane systems in the cellular components (Figure 9c,d).

**Figure 10:**
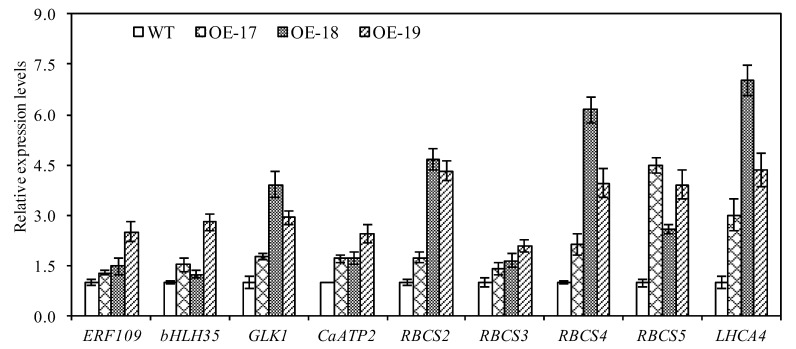
The relative expression of the selected differentially expressed genes in rice in response to salt stress.

To identify the expression profiles of the up-regulated differentially expressed genes that are involved in metabolic cycles and photosynthetic process, a total of nine genes with up-regulation were specifically detected by a quantitative RT-PCR procedure under salt stress (primers: Table S1; genes: Table S2). The qRT-PCR data in Figure 10 shows that the three transgenic lines with up-regulated genes had differential expression levels of the co-expressed unigenes, such as *CaATP2* (plant-type II Ca^2+^ATPases), *RBCS* (ribulose bisphosphate carboxylase small chain-*RuBisCO*), and *LHCA4* (light-harvesting complex I chlorophyll a/b binding protein 4). Namely, *LHCA4*, *RBCS*2, *RBCS4*, and *RBCS5* demonstrated remarkable up-regulations in response to a salt stress of 100 mmol·L^−1^ of NaCl. The data also revealed that only the OE-19 line significantly up-regulated the expression of three transcription factors: *ERF* (ethylene response factor), *bHLH35* (basic helix-loop-helix 35), and *GLK1* (Golden-like 1 transcription factor). Both the OE-18 and OE-19 lines remarkably up-regulated the *GLK1*, *RBCS*2, *RBCS4* and *LHCA4* unigenes under salt stress compared to the other lines. Our data seems to be identical to the presented results of the most enriched gene GO terms (Table S3).

## 3. Discussion

Both plasma membrane intrinsic proteins (PIP) and tonoplast intrinsic proteins (TIP) have regulatory roles in maintaining the osmotic potential in response to abiotic stresses [12], and they are especially involved in the allocation of water and photosynthetic metabolites by performing rapid water transport in the roots [13]. In the case of osmotic stress, the *McTIP1;2* gene in *Mesembryanthemum crystallinum* is quickly transferred from the vacuolar membrane to the cytoplasm with a redistribution of intracellular ions and plays a regulatory role in maintaining the osmotic balance in plants [14]. The knockdown of the *AtPIP1;2* gene leads to more than 50% decrease in osmotic conductivity in mesophyll cells [15]. Higher accumulation of the *TsTIP1;2* transcripts exhibits a secondary response to salt stress and it beneficially triggers changes in the physiological metabolism in rice [10]. In our study, the higher accumulation of the *TsPIP1;1* transcripts in the transgenic lines might have played an important role in promoting the conductance and permeability of water in rice under salt stress, because two rice homologous of the *TsPIP1;1* gene, the myc-tagged *OsPIP1;1* and *OsPIP1;2*, did not promote water permeability in a yeast system under salt stimulus [16]. This study also confirmed that the overexpression of the *OsPIP1;1* gene not only mitigates water conductance, but also inhibits the expression of the other endogenous aquaporins in the plant [17].

Generally, the transportation and permeability of water in plants accompanies a translocation or distribution of metabolites and Na/K ions, and salt stress easily induces ion homeostasis and osmotic adjustment. The higher accumulation of soluble sugar, proline, and K ion alleviates salt damage to plants by regulating the osmotic potential or lowering the Na/K ratio in plant cells [18]. Overexpression of the *TsMIP6* gene increases the contents of soluble sugar and proline in the shoots of the transgenic rice under salt stress, but the involvement mechanism of *TsMIP* in the physiological metabolism is not elucidated completely [19]. The transgenic *Arabidopsis* overexpressing the *MaPIP1;1* gene increases the K/Na ratio and improves the salt tolerance [20]. Transgenic *Arabidopsis* that overexpresses the *MzPIP2;1* gene shows an enhanced salt tolerance by accumulating more K [21]. In our study, the overexpression of the *TsPIP1;1* gene significantly increased the K content and lowered the Na/K ratio in the transgenic rice, thus playing an important regulatory role in decreasing the osmotic potential. The changes in osmotic potential are generally related to the influx or efflux of Na and K ions in the root tips. The overexpression of the *SlTIP2;2* gene in the transgenic *Arabidopsis* significantly decreases the K efflux and Na influx in the root tips under salt stress [22]. *AtLOS5* overexpression in transgenic maize enhances the net Na efflux in the roots but decreases the net K efflux [23]. In this study, the efflux profiles of Na and K ions in the roots of the transgenic lines suggested that the transgenic lines discharge more Na and accumulate more K, implying that flux changes in Na and K ions in the transgenic plants might participate in the homeostasis modulation of ions in the roots. Except for Na-trigered damage, MDA—an indicator of stress-induced peroxidation of membrane lipids—is widely used for evaluating plant cell injuries [24]. The transgenic banana that overexpresses the *MusaPIP2;6* gene lowers the membrane damage by reducing the accumulation of MDA under salt stress [25]. Our data suggested that the expression of the *TsPIP1;1* gene reveals a positive regulatory role in lowering the MDA content in transgenic rice.

Salt stress not only effects the accumulation of physiological metabolites and ions, but also severely inhibits photosynthesis by directly destroying the plastid components in the chloroplast [26]. *AQP* genes can reduce the inhibition of salt stress-triggered photosynthesis by controlling the transport of water and metabolic substances. Constitutive overexpression of the *NtAQP1* gene in tobacco and tomatoes confers salt tolerance by increasing the net rates of photosynthesis and mesophyll CO_2_ conductance [27]. Under saline conditions, the net assimilation, stomatal conductance, and transpiration rates are significantly improved by involvement of the *GmPIP1;6* gene in the transgenic soybean [28]. Chlorophyll, an essential pigment in chloroplasts, is one of the most important indicators of photosynthesis strength. In this study, higher levels of chlorophyll in the transgenic rice promoted greater photosynthetic rates, stomatal conductance, intercellular CO_2_ concentrations, transpiration rates, and water use efficiency in response to a salt stress of 200 mmol·L^−1^ NaCl, suggesting that the *TsPIP1;1* gene participates in the regulation of photosynthesis. Plastids and chloroplasts are essential for the photosynthesis of plants. Additionally, the most enriched GO terms of the up-regulated genes in the OE-17 line responded to external stressful stimulus (Figure 9b), and the most enriched GO terms of the up-regulated genes in both the OE-18 and the OE-19 lines largely responded to chloroplasts or plastids (Figure 9c,d), indicating that the transgenic lines have diverse adaptive responsive mechanisms to salt stress. The OE-17 line predominantly exhibited up-regulation of the differentially expressed genes in biological processes in the responses to stimuli, but the up-regulated differentially expressed genes in the transgenic OE-18 line predominantly responded to plastids and chloroplasts in the cellular component and primary metabolic process. Like the OE-18 line, the transgenic OE-19 line primarily showed up-regulation of differentially expressed genes in the cellular components. These results show that the up-regulation of differentially expressed genes due to salt stimuli, plastids, and chloroplasts influences the photosynthetic process and translocations of the physiological metabolites in plants. In this study, the major transcription factors (*C2H2*, *AP2/ERF*, *bHLH*, *WRKY*, and *MYB*) or Ca^2+^-dependent protein kinase (*CDPKs*) of the up-regulated or down-regulated differentially expressed genes in the transgenics were demonstrated (Table S3) and shown to function in signal transduction pathways in response to abiotic stresses by positively or negatively regulating the expressions of the target genes [29]. For example, the ectopic expression of the *ThZF1* gene in *Arabidopsis* mutant *azf2* improves the phenotype in response to salt stress [30]. Wang et al. (2008) reported that the *OsDREB1F* gene, a member of the *AP2/ERF* subfamily, confers an enhanced salt tolerance in transgenic *Arabidopsis* and rice [31]. Overexpression of the *bHLH* gene improves salt tolerance in wild rice [32] and the *bHLH* transcription factors were also confirmed to play a regulatory role in facilitating K uptake during stomatal openings [33]. In our study, the remarkable up-regulation of the transcription factor *bHLH35* in the OE-19 line might promote the uptake and translocation of K in rice, thus altering the ratio of Na/K. Zhou et al. (2015) found that the overexpression of the *GhWRKY34* gene significantly raises the seed germination rate, root length, and chlorophyll contents in the transgenic *Arabidopsis* under salt stress [34]. The up-regulation of the *TaMYB3R1* gene under salt stress participates in responses to abiotic stresses [35]. Saijo et al. (2000) reported that overexpression of a Ca^2+^-dependent protein kinase—the *OsCDPK7* gene—enhances salt tolerance by inducing some stress-responsive genes in response to salinity stress [36]. Later findings confirmed that the passive transport of K and Na ions in plants is mainly performed by the outward rectifying K ion channel under salt stress, and the external Ca^2+^ might alleviate salt damage to the plants [37]. Kazi et al. (2013) reported that the *CaATP2* (plant-type II Ca^2+^ATPases) gene plays an essential role in the stomatal opening or closing, as well as in calcium signaling and stress responses [38]. Meanwhile, the most enriched pathways in transgenic rice are chiefly represented by the photosynthesis-antenna protein, glyoxylate, dicarboxylate metabolism, porphyrin, and chlorophyll metabolism (Figure S1), indicating that these important metabolites participate in the tricarboxylic acid cycle and the photosynthetic process. Our study showed that the photosynthesis-antenna protein, porphyrin, and chlorophyll play vital roles in plant photosynthesis by absorbing light and transferring solar energy via pigment-binding proteins [39].

Photosynthesis is usually regulated by critical subunits of ribulose bisphosphate carboxylase (Rubisco) that catalyze the entry of carbon dioxide into the photosynthetic metabolism. This study showed that photosynthesis is not limited by *Rubisco* only if plants encounter severe or long-term salt stress [40]. A higher chloroplastic carbon dioxide concentration (Cc) activates *Rubisco* in some plant species under abiotic stress, thus greatly promoting photosynthetic rates [41,42]. Photosynthesis is closely related to light capture and the efficient energy transfer occurring in photosynthetic reaction centers. The light-harvesting chlorophyll a/b-binding proteins associated with photosystem II are the most abundant pigment–protein complex in leaves and they function in capturing light and transferring energy [43]. In our study, the transgenic rice significantly increased the intercellular CO_2_ concentration and stomatal conductance, and led to greater chlorophyll accumulation with apparent up-regulation of the *RBCS* (ribulose bisphosphate carboxylase small subunit), *LHCA4* (light-harvesting chlorophyll a-binding), and *CaATP2* genes after short-term salt stress, showing that the up-regulation of these differentially expressed genes in enriched gene GO terms plays an important role in promoting photosynthesis by altering the allocations of photosynthetic metabolites in the transgenic rice.

## 4. Materials and Methods

### 4.1. Plant Culture and Treatments

Seeds of both the wild-type and the T3 generation rice of the transgenic lines, OE-17, OE-18, and OE-19, were sterile-planted on the Murashige and Skoog (MS) solid medium containing different NaCl concentrations (0 or 100 or 200 mmol·L^−1^) and cultured in a growth chamber at 28 °C with 75% humidity under a 16 h light intensity of 6000 lux and at 23 °C for 8 h in the dark for two weeks. Meanwhile, a hydroponics experiment was performed under the same levels of salt stress. Briefly, the T3 generation seeds of the transgenic lines and the wild-type were cultured in water in an incubator at 30 °C under darkness for 3 days, and the rice seedlings were transplanted and hydroponically cultured with a Hoagland solution (pH: 5.5–6.5) in a greenhouse under an illumination intensity of 8000–10,000 lux for 12 h with 75% relative humidity for four weeks. Furthermore, the culture solutions were renewed in intervals of 3 days. Then, the five-week-old seedlings were separately cultured in solutions of 0, 100, or 200 mmol·L^−1^ NaCl for 7 days under the same environmental conditions. The plant samples with three biological replicates were collected for physiological measurements, and the remained rice seedlings were continuously cultured in the Hoagland solutions to harvest grains.

### 4.2. Plant Transformation and Subcellular Localization

A full-length cDNA fragment of the *TsPIP1;1* gene (accession no. JX133234) was amplified by PCR using a pair of specific primers TsPF and TsPR (Table S1) and inserted into the pCAMBIA1304 expression vector under the control of the 35S promoter, and then the generating fusion constructs were transferred into the rice cultivar Kitaake (*Oryza sativa* L. ssp. japonica) using an *Agrobacterium* GV1301-mediated method. The transgenic rice of T3 generation with stable heredity, OE-17, OE-18, and OE-19, were obtained from three years of continuous screening for further analyses. Similarly, the cDNA fragments of the *TsPIP1;1* gene were amplified by PCR with specific primers (Table S1) and inserted into the *BamH*I and *Hind*III sites onto a 16318hGFP vector harboring a 35S promoter, thereby obtaining the fusion construct of the *TsPIP1;1::GFP*. Both the *TsPIP1;1::GFP* construct and the 16318hGFP vector were separately transformed into the onion epidermal cells by particle bombardment technology (Bio-Rad, Richmond, VA, USA). The bombarded onion epidermal cells were cultured under darkness at 25 °C overnight, and the transient expressions of the GFP were observed with an LSM700 confocal laser microscope (Carl Zeiss AG, Oberkochen, Germany) and all images were processed with ZEN 2009 Light Edition software (Carl Zeiss Microscopy GmbH, Munich, Germany).

### 4.3. RNA Extraction and qPCR Analyses

The total RNA of the roots and shoots of rice was separately prepared with an RNA Easy Kit (TransGen Biotech, Beijing, China). A total of 2 μg of RNA was used for cDNA synthesis by RT-PCR using a one-step gDNA removal and a cDNA synthesis supermix (TransGen Biotech, Beijing, China). A total of 1 μL of cDNA was added to a reaction solution of 20 μL containing 10 μL of Top Green qPCR Supermix and 0.2 μM of the specific primers (Table S1) and quantitative RT-PCR was performed with a light cycler system (iQ5, Bio-Rad, Richmond, VA, USA) under the following conditions: 94 °C for 10 min with 40 cycles of 94 °C for 5 s, 60 °C for 15 s, and 72 °C for 20 s. Based on the expression folds of the reference gene *UBQ3* (accession no. Os02g0634800), the relative expression levels of the target genes were calculated using the 2^−ΔΔ*C*t^ formula [44].

### 4.4. Morphological Parameters and Physiological Measurements

Both solid medium-culture and hydroponics of rice were performed to profile the changes of the root morphology, physiological metabolites, shoot heights, root lengths, fresh weights of the shoots and roots, seed ripening rates, and grain yield per plant. A total of 0.5 g of fresh leaves was extracted for the measurements of chlorophyll, proline, solute sugar, and malondialdehyde (MDA) [19]. Measurements of Na and K in the shoots were performed by atomic absorption [45]. The relative water contents were determined as previously described [10]. The photosynthetic parameters of leaves were determined using a Li-6400XT portable photosynthesis system (LICOR Biosciences, Lincoln, NE, USA). The leaf sap was generated by squeezing the frozen leaves at −80 °C using a syringe tube of 25 mL, and the osmotic potential was determined by an osmotic pressure dew point meter (Wescor 5520, Logan, UT, USA). All the data were statistically analyzed from three biological replicates.

### 4.5. Measurement of Na and K Ion Flux

The seeds of both transgenic and wild-type rice were germinated in the water solution at 30 °C in dark for 2 days and then transferred to the Hoagland solution and cultured for 7 days. The seedlings were treated with 0 or 200 mmol·L^−1^ NaCl for 24 h to determine the net fluxes of Na and K ions in the root elongation zone (500 μm, apart from the root tip). The calibrated electrodes were moved to the root tips in the range of 0.3–0.5 Hz for 8 min with the NMT technique of USA Younger (Xuyue Sci. & Tech. Co. Lit., Beijing, China), and the net fluxes of Na and K were determined with Mage Flux software (available online: http://www.youngerusa.com/mageflux).

### 4.6. Analyses of Transcriptome Sequencing

To eliminate possible statistical errors, five leaves from the same position in each sample were uniformly mixed to extract the total RNA. The quality of total RNA was assessed with Nanodrop 2000, and a total of 10 μg of the RNA fraction of 0.1–4.0 μg·μL^−1^ was used for the synthesis of cDNA. Then, the generated cDNA was purified and decorated with AMPure XP beads. Finally, the cDNA library was sequenced on the Illumina Hiseq^TM^ 2500 platform. All the raw data were converted into the FASTAQ format, deposited in the NCBI Sequencing Read Archive Database, and blasted against publicly available sequence databases with the BLAST program. RPKM (reads per kilobase of exon model per million mapped reads) was used to identify differentially expressed genes (DEGs) [46]. The absolute values with log_2_ (fold change) > 1 and *p*-adjusted < 0.005 were used as thresholds to judge significant differences in gene expression. The differentially expressed genes were confirmed by assembly combination between the wild-type and the transgenic lines and analyzed by the Gene Ontology (GO) (available online: http://www.geneontology.org/) and Kyoto Encyclopedia of Genes and Genomes (KEGG pathways).

### 4.7. Bioinformatics Analysis and Statistical Methods

The structural characteristics of the *TsPIP1;1* gene were profiled by the map viewer in the website at NCBI. The transmembrane helices of the TsPIP1;1 protein were predicted by TMHMM Server 2.0 version. All of the data represent at least three biological replicates, and were analyzed with Microsoft Excel 2013 (Microsoft Corporation, Redmond, WA, USA), and one way ANOVA was performed by using a LSD method of Post hoc-tests (SPSS 19.0 version, SPSS Inc., Chicago, IL, USA).

## 5. Conclusions

Overexpression of the *TsPIP1;1* gene modulated the transport of water, accumulation profiles of metabolites, homeostasis of Na/K ions, and photosynthesis in the transgenic rice, and triggered the up-regulation of the differentially expressed genes, thereby contributing to the beneficial allocation of photosynthetic metabolites and photosynthesis in plants in response to high salinity conditions. The *TsPIP1;1* gene conferred an enhanced salt tolerance by participating in osmotic potential modulation and photosynthesis promotion in transgenic rice.

## Figures and Tables

**Table 1:** The accumulation profiles of Na^+^ and K^+^ in the shoots of rice.

NaCl Treatment	Lines	Na^+^ Content (mg/g, DW)	K^+^ Content (mg/g, DW)	Na^+^/K^+^ Ratio
0 mM	WT	2.44 ± 0.33	16.50 ± 1.02	0.15 ± 0.02
	OE-17	2.53 ± 0.42	16.73 ± 0.59	0.15 ± 0.02
	OE-18	2.58 ± 0.17	17.51 ± 0.69	0.15 ± 0.01
	OE-19	2.53 ± 0.26	17.77 ± 0.65	0.14 ± 0.02
100 mM	WT	14.42 ± 0.42	8.61 ± 0.61	1.68 ± 0.08
	OE-17	14.01 ± 0.66	10.03 ± 0.72	1.40 ± 0.06 *
	OE-18	14.08 ± 0.80	10.28 ± 1.31 *	1.38 ± 0.18 *
	OE-19	13.54 ± 0.95	9.92 ± 0.44	1.37 ± 0.12 **
200 mM	WT	27.90 ± 0.65	5.28 ± 0.72	5.35 ± 0.58
	OE-17	25.21 ± 1.27 **	6.70 ± 0.95 *	3.80 ± 0.35 **
	OE-18	25.62 ± 1.39 *	7.80 ± 0.80 **	3.32 ± 0.48 **
	OE-19	25.60 ± 0.89 *	7.88 ± 0.54 **	3.26 ± 0.33 **

The error bars indicate the ±SD of five biological replicates. WT represents wild-type; OE-17, OE-18, and OE-19 represent the transgenic rice lines, respectively. DW: dried weight; * and ** indicate significant differences at the *p* < 0.05 level and *p* < 0.01 level, respectively (*LSD*-test).

**Table 2:** Changes in the photosynthetic parameters of rice.

NaCl	Lines	Pn μmol·m^−2^·s^−1^	Gs mol·m^−2^·s^−1^	Ci μmol/mol	Tr mmol m^−2^·s^−1^	Wue μmol/mmol
0 mM	WT	12.91 ± 0.09	0.44 ± 0.01	165.85 ± 0.57	4.22 ± 0.13	3.06 ± 0.09
	OE-17	13.11 ± 0.11 *	0.43 ± 0.01	166.59 ± 2.55	4.17 ± 0.08	3.14 ± 0.03 *
	OE-18	13.07 ± 0.31	0.45 ± 0.01	166.23 ± 1.48	4.25 ± 0.10	3.07 ± 0.08
	OE-19	14.04 ± 0.16 **	0.49 ± 0.01 **	164.37 ± 2.48	4.36 ± 0.11 **	3.22 ± 0.08 **
100 mM	WT	6.13 ± 0.49	0.30 ± 0.01	142.91 ± 0.83	3.70 ± 0.07	1.66 ± 0.13
	OE-17	6.88 ± 0.55 **	0.32 ± 0.01 **	144.92 ± 0.65 **	3.83 ± 0.25	1.80 ± 0.09 **
	OE-18	6.89 ± 0.19 **	0.31 ± 0.01 *	143.49 ± 0.25 *	3.61 ± 0.18	1.91 ± 0.08 **
	OE-19	7.43 ± 0.37 **	0.35 ± 0.01 **	144.86 ± 0.47 **	3.92 ± 0.09 **	1.90 ± 0.12 **
200 mM	WT	3.45 ± 0.29	0.21 ± 0.01	117.42 ± 1.19	2.64 ± 0.11	1.31 ± 0.10
	OE-17	4.30 ± 0.27 **	0.27 ± 0.01 **	129.84 ± 0.61 **	2.96 ± 0.04 **	1.45 ± 0.08 **
	OE-18	4.21 ± 0.22 **	0.24 ± 0.01 **	133.43 ± 1.49 **	2.95 ± 0.05 **	1.43 ± 0.07 **
	OE-19	4.94 ± 0.30 **	0.29 ± 0.02 **	132.99 ± 0.63 **	3.13 ± 0.32 **	1.59 ± 0.12 **

Pn: Photosynthetic rates; Gs: stomatal conductance; Ci: intercellular CO_2_ concentration; Tr: transpiration rate; Wue: water use efficiency. Error bars indicate ±SD of five biological replicates. WT represents wild-type; OE-17, OE-18, and OE-19 represent the transgenic rice lines. * and ** indicate significant differences at the *p* < 0.05 level and at the *p* < 0.01 level, respectively (*LSD*-test).

## Supplementary Materials

Supplementary materials can be found at http://www.mdpi.com/1422-0067/19/8/2229/s1.

## Abbreviations

PIP	Plasma membrane intrinsic proteins
TIP	Tonoplast intrinsic proteins
NIP	Nod26-like intrinsic proteins
SIP	Small and basic intrinsic proteins
XIP	Uncharacterized intrinsic proteins
DEG	Differentially expressed genes
MDA	Malondialdehyde
NMT	Non-invasive micro-test technique
MS	Murashige and Skoog medium
*ERF109*	Ethylene response factor109
*bHLH35*	Basic helix-loop-helix 35
*GLK1*	Golden-like 1 transcription factor
*CaATP2*	Plant-type II Ca^2+^ATPases
*RBCS*	Ribulose bisphosphate carboxylase small chain
*LHCA4*	Light-harvesting complex I chlorophyll a/b binding protein 4
