# Supplementary material for: Ectopic Expression of a Thellungiella salsuginea Aquaporin Gene, TsPIP1;1, Increased the Salt Tolerance of Rice
Source: Int J Mol Sci. 2018 Jul 30;19(8):2229. doi: 10.3390/ijms19082229 (PMC6122036; doi:10.3390/ijms19082229)
Supplement: Supplementary file 1 [file ijms-19-02229-s001.pdf]

Supplementary data Table S1. Gene specific primers used in this study

Item	Gene Name	Gene ID number	Primer sequence
Vector construction	TsPIP1::GFP	JX133234	F: 5’-AAGCTTATGGACGGCAAAGAAGA-3’
			R: 5’-GGATCCGCTCTTGAAAGGAATG-3’
RT-PCR	*TsPIP1;1*	JX133234	F: 5’-GTACACCTGGCGACAATTCC-3’
			R: 5’-GGTCCAACCCAGAAAATCCA-3’
	ERF109	Os09G0457900	F: 5’-GGATAGATGAGATTCCTGTGAG-3’
			R: 5’-GTTGAGTTCTGCATTGAGGTC-3’
	bHLH35	Os04g0301500	F: 5’-GTCATTTTTGCGACACCCCA-3’
			R: 5’-CCTCGCAAGAAAACAACCACC-3’
	GLK1	Os06G0348800	F: 5’-AGCTGCGAGATTTCCTGCTC-3’
			R: 5’-ATAGCTGCGTCGATGCTCTC-3’
	CaATP2	Os03g0203700	F: 5’-GGAATGCGGCATTTTGACTG-3’
			R: 5’-CTTGGTTCTGAAATCCGGTCCT-3’
	RBCS2	Os12g0274700	F: 5’-CAACTAAGCCGTCATCGTC-3’
			R: 5’-CCTCACCCAACAACATATAGTC-3’
	RBCS3	Os12g0291100	F: 5’-CAATGGCCTCTGCTCTTAAC-3’
			R: 5’-GCCGACGAATTTATCAGATATG-3’
	RBCS4	Os12g0292400	F: 5’-TACTACAACTATCGGTCTATCGTTC-3’
			R: 5’-TGTCGACGGATTCAGATATG-3’
	RBCS5	Os12g0291400	F: 5’-AGCTTTGAACGTTCGAATC-3’
			R: 5’-CATACACAGCTGATGTTGACAC-3’
	LHCA4	Os08G0435900	F: 5’-CTACCTTCCTAGGGCAATCC-3’
			R: 5’-TTGAGGTAGGTGGGAGAAGG-3’
	UBQ3	Os02g0634800	F: 5’-AAGGCATGGCTGATTCATGT-3’
			R: 5’-CAGATGTTCACATGGTGACAGTA-3’

Supplementary data Table S2. Expression profiles of up- and down-regulated differentially expressed genes.

Gene name	Gene ID	log_2fold	Corrected P-Value	Up or down regulation
C2H2	Os03G0820400	-1.5215	3.9322E-16	down
	Os03G0786400	1.9505	2.1642E-07	up
	Os07G0581366	1.2388	0.000097158	up
	Os02G0775600	1.2769	0.001385	up
AP2/ERF	Os02G0752800	-1.0239	0.00065716	down
	Os04G0546800	-1.1621	9.6123E-09	down
	Os06G0166400	-1.0514	0.000086157	down
	Os08G0474000	3.101	0.00094189	up
	Os09G0457900	3.0104	1.3651E-10	up
WRKY	Os05G0322900	1.1052	0.000053685	up
	Os05G0474800	-1.199	2.2212E-19	down
	Os09G0417600	3.2151	3.1913E-23	up
	Os09G0417800	3.4388	0.00015673	up
	Os01G0584900	-2.1061	4.4308E-08	down
	Os01G0656400	-2.1875	9.742E-16	down
	Os02G0462800	-1.4191	7.7888E-06	down
bHLH	Os01G0566800	2.4709	0.00076773	up
	Os01G0575200	-1.059	0.00020205	down
	Os03G0741100	1.3511	3.18E-23	up
	Os03G0782500	1.3074	0.00021517	up
	Os04G0301500	2.8469	9.4279E-18	up
	Os09G0455300	-1.4075	0.0013425	down
MYB	Os01G0874300	-1.1476	0.00016325	down
	Os02G0641300	-2.3503	9.0618E-07	down
	Os06G0348800	1.205	1.6305E-15	up
	Os06G0637500	-1.1787	0.000043914	down
	Os12G0564100	1.7919	1.1506E-13	up
CDPK	Os01G0557500	-1.2106	2.7248E-09	down
	Os01G0955100	-1.2151	1.5419E-17	down
	Os02G0122600	-1.6934	0.000016445	down
	Os03G0128700	1.1971	0.00021544	up
	Os03G0203700	2.9559	1.2509E-06	up
	Os03G0319300	-1.0086	1.1761E-18	down
	Os06G0172200	-1.1592	1.4453E-06	down
	Os07G0678300	1.4697	3.4663E-06	up
	Os08G0441100	-1.509	8.6226E-07	down

Supplementary data Table S3. Profiles of the differentially expressed genes specifically involved in the transcription regulation or photosynthetic system in the KEGG pathways.

Gene name	Gene ID	Log2fold	P value	Q value	Function annotation	Transgenic line
RBCS2	OS12G0274700	1.9532	5.95E-168	1.25E-164	ribulose bisphosphate carboxylase small chain, chloroplastic	OE-17
RBCS3	OS12G0291100	1.025	5.62E-80	3.72E-77	ribulose bisphosphate carboxylase small chain, chloroplastic	OE-17
RBCS4	OS12G0292400	1.0522	2.33E-33	5.42E-31	ribulose bisphosphate carboxylase small chain, chloroplastic	OE-17
RBCS5	OS12G0291400	1.8712	2.93E-43	1.01E-40	ribulose bisphosphate carboxylase small chain, chloroplastic	OE-17
LHCA4	OS08G0435900	1.3138	1.63E-15	1.35E-13	light-harvesting complex I chlorophyll a/b binding protein 4	OE-17
GLK1	OS06G0348800	1.9077	4.60E-56	7.57E-54	probable transcription factor	OE-18
RBCS2	OS12G0274700	2.562	0	0	ribulose bisphosphate carboxylase small chain, chloroplastic	OE-18
RBCS3	OS12G0291100	1.9574	0	0	ribulose bisphosphate carboxylase small chain, chloroplastic	OE-18
RBCS4	OS12G0292400	2.1745	3.44E-236	3.21E-233	ribulose bisphosphate carboxylase small chain, chloroplastic	OE-18
RBCS5	OS12G0291400	1.3714	1.98E-24	1.35E-22	ribulose bisphosphate carboxylase small chain, chloroplastic	OE-18
LHCA4	OS08G0435900	4.4298	0	0	light-harvesting complex I chlorophyll a/b binding protein 4	OE-18
ERF109	OS09G0457900	3.0104	1.78E-12	1.37E-10	ethylene response factor 109	OE-19
bHLH35	OS04G0301500	2.8469	6.73E-20	9.43E-18	basic helix loop-helix 35	OE-19
GLK1	OS06G0348800	1.205	1.37E-17	1.63E-15	probable transcription factor	OE-19
CaATP2	OS03G0203700	2.9559	2.82E-08	1.25E-06	Calcium-transportin ATPase2	OE-19
RBCS2	OS12G0274700	2.0397	4.20E-202	1.51E-198	ribulose bisphosphate carboxylase small chain, chloroplastic	OE-19
RBCS3	OS12G0291100	1.4257	1.52E-194	4.78E-191	ribulose bisphosphate carboxylase small chain, chloroplastic	OE-19
RBCS4	OS12G0292400	1.3435	4.05E-65	3.40E-62	ribulose bisphosphate carboxylase small chain, chloroplastic	OE-19
RBCS5	OS12G0291400	1.8271	9.27E-44	3.77E-41	ribulose bisphosphate carboxylase small chain, chloroplastic	OE-19
LHCA4	OS08G0435900	1.7284	5.90E-32	1.63E-29	light-harvesting complex I chlorophyll a/b binding protein 4	OE-19

Supplementary data Figure S2. The distributions of the most enriched GO terms in the three KEGG pathways.

A

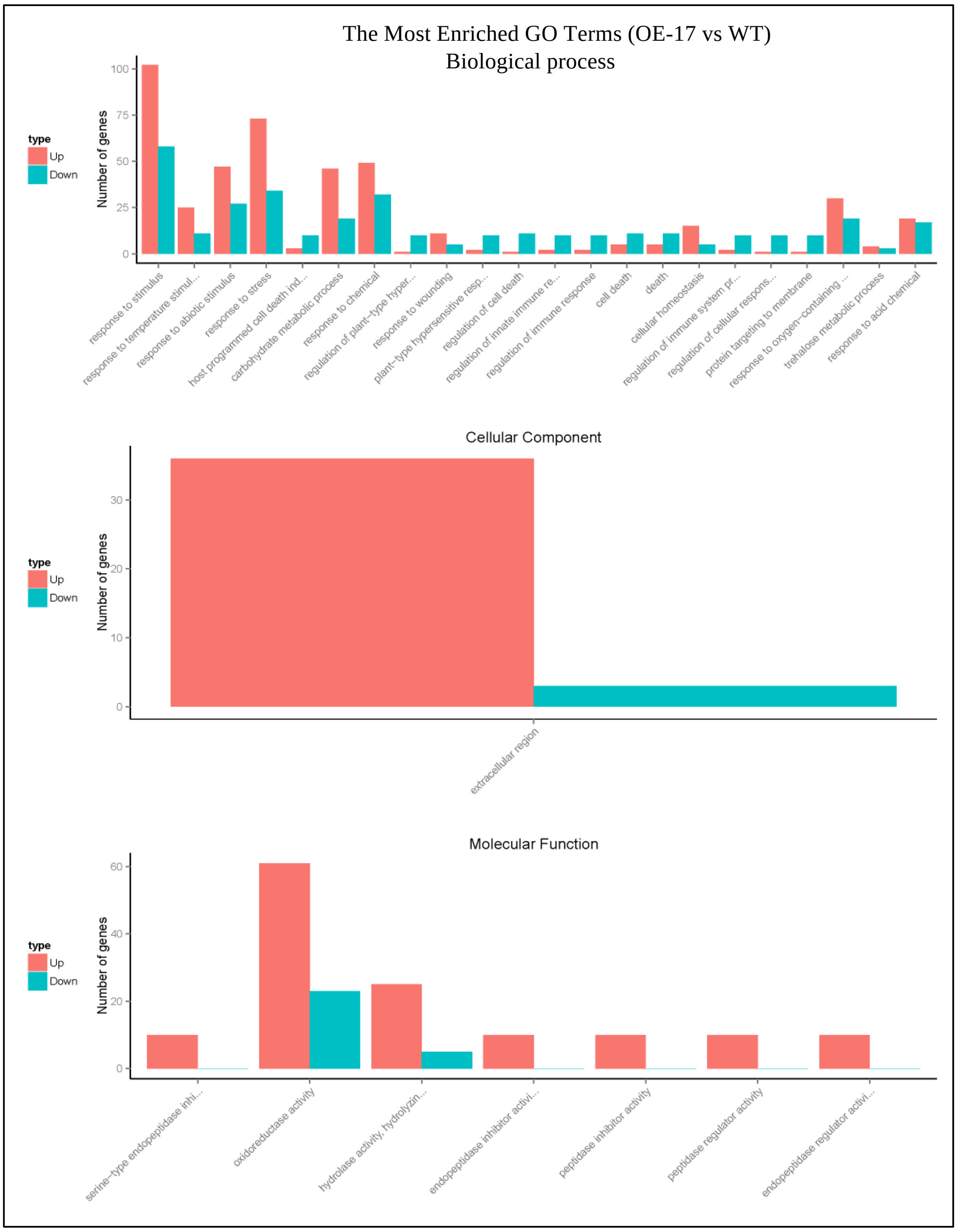

B

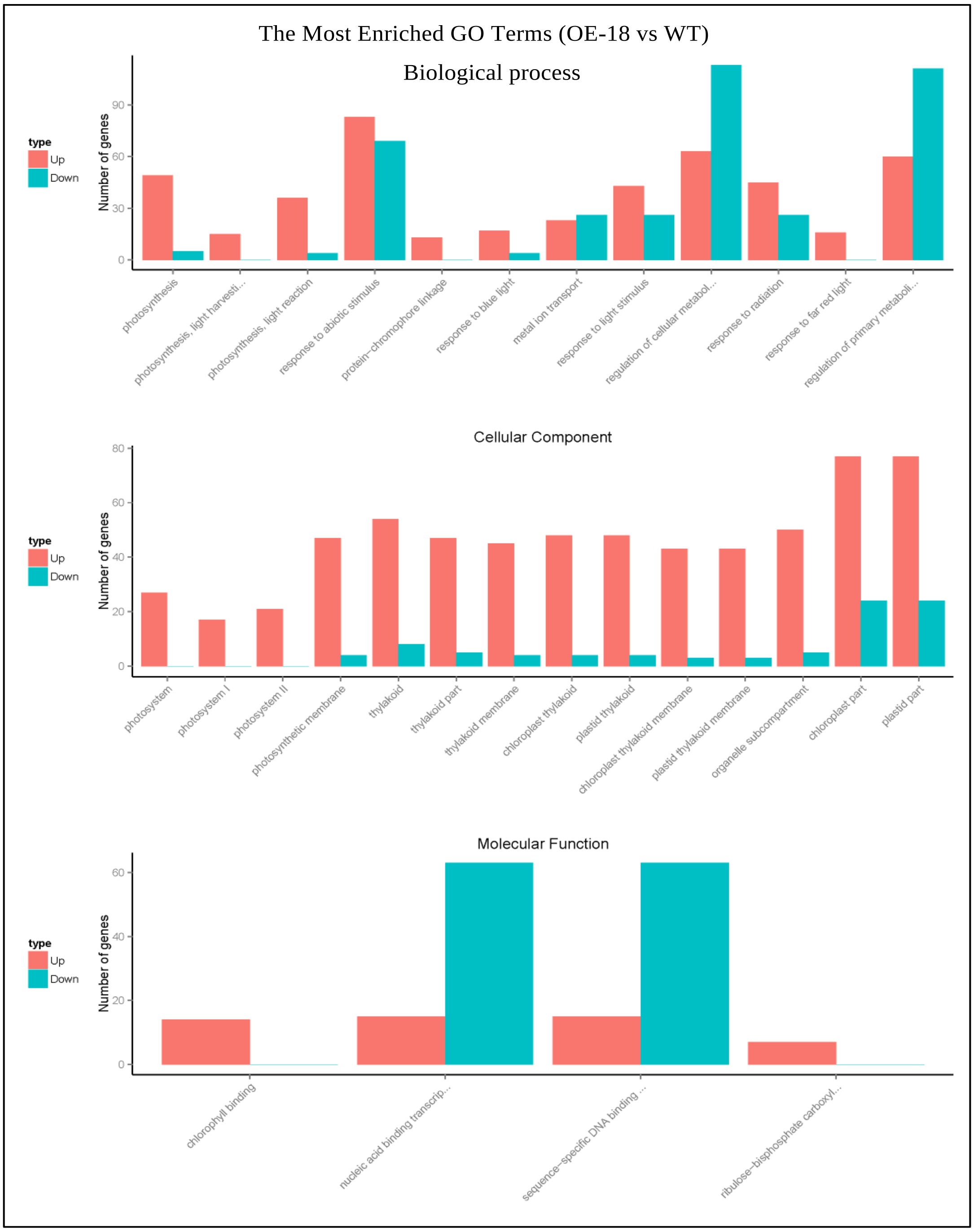
The Most Enriched GO Terms (OE-18 vs WT)
Biological process
type
Up
Down
Number of genes
0
30
60
90
photosynthesis
photosynthesis, light harvesti...
photosynthesis, light reaction
response to abiotic stimulus
protein–chromophore linkage
response to blue light
metal ion transport
response to light stimulus
regulation of cellular metabol...
response to radiation
response to far red light
regulation of primary metaboli...
Cellular Component
0
20
40
60
80
photosystem
photosystem I
photosystem II
photosynthetic membrane
thylakoid
thylakoid part
thylakoid membrane
chloroplast thylakoid
plastid thylakoid
chloroplast thylakoid membrane
plastid thylakoid membrane
organelle subcompartment
chloroplast part
plastid part
Molecular Function
0
20
40
60
chlorophyll binding
nucleic acid binding transcrip...
sequence–specific DNA binding ...
ribulose–bisphosphate carboxyl...

C

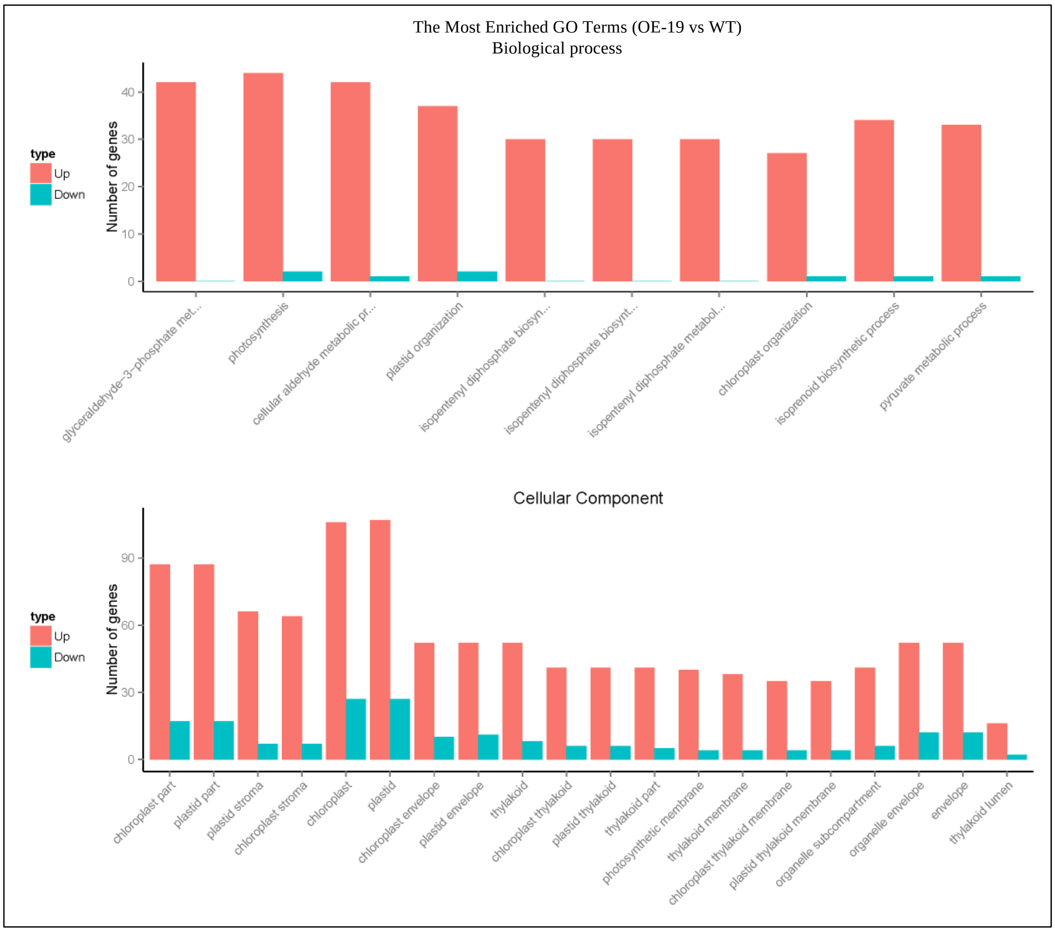

(A) The most enriched GO terms in the OE-17 line; (B) The most enriched GO terms in the OE-18 line; (C) The most enriched GO terms in the OE-19 line, and no enriched GO terms in molecular function were observed.
